# Endoaesthetic Management of Type II Dens Invaginatus Associated With Root Perforation and Apical Abscess: A Case Report

**DOI:** 10.1155/crid/2909252

**Published:** 2025-04-28

**Authors:** Alessandro Moreira Freire, Orlando Aguirre Guedes, Lara Borges de Deus, Paulo Eduardo Mafra, Gustavo Silva Chaves, Karolina Kellen Matias, Juliano Gonçalves Miguel, Erica Eugênia Javarez Freire, Daniel de Almeida Decurcio

**Affiliations:** ^1^Department of Endodontics, Alfredo Nasser University Center, Goiânia, Goiás, Brazil; ^2^Department of Endodontics, Evangelical University of Goiás, Anápolis, Goiás, Brazil; ^3^Department of Stomatology, Federal University of Goiás, Goiânia, Goiás, Brazil; ^4^Brazilian Dental Association, Goiânia, Goiás, Brazil; ^5^Department of Oral Biology, Pontifical Catholic University of Goiás, Goiânia, Goiás, Brazil

**Keywords:** aesthetics, apical abscess, dens invaginatus, nonsurgical endodontic treatment

## Abstract

Dens invaginatus (DI) is a developmental anomaly that affects teeth. This case report demonstrates the successful endoaesthetic management of a Type II DI in the maxillary right lateral incisor (Tooth 12). A 12-year-old female patient presented with pain and swelling in Tooth 12, which had previously been diagnosed with DI and accessed endodontically. The patient was also concerned about the aesthetic appearance of her anterior teeth. Clinical, radiographic, and tomographic findings confirmed Oehler's type II DI, with root perforation and an apical abscess in Tooth 12, as well as pulp necrosis in Tooth 13. Nonsurgical root canal treatment was recommended for both teeth, with additional root perforation repair for Tooth 12. Multiple visits were required for nonsurgical endodontic therapy. During the initial visit, the perforation was identified and sealed, followed by chemomechanical preparation and placement of calcium hydroxide paste. Ten months later, the root canals were filled. After endodontic treatment, the patient underwent in-office bleaching, followed by composite resin veneers on the upper anterior teeth, using the preformed metal matrix technique. This resulted in excellent aesthetic outcomes. At the 2-year follow-up examination, the patient remained asymptomatic, and radiographic assessment showed complete resolution of the periapical lesion. This case highlights the importance of multidisciplinary collaboration, precise treatment planning, and patient-centered care in achieving optimal endoaesthetic results in complex cases.

## 1. Introduction

Dens invaginatus (DI) is a developmental dental anomaly characterized by the invagination of the enamel organ into the dental papilla before the mineralization phase (morphodifferentiation stage) [1]. The incidence of this anomaly ranges from 0.04% to 12% [2–4], with approximately 42% of cases occurring in the permanent maxillary lateral incisors [5–7].

The most widely accepted classification of DI was introduced by Oehlers [8]. This classification describes a total of four subtypes (I, II, III-A, and III-B) based on the extent of the invaginated tissue [8]. In Type I, the invagination is confined to the coronal part of the tooth. In Type II, the invagination extends apically beyond the cementoenamel junction, terminating in a blind sac. In Type III, the invagination extends through the root and connects with the periodontal ligament laterally or at the apical foramen [8]. Pulp necrosis and apical periodontitis are more frequently associated with type III DI due to the extension of the invagination to the apical foramen. However, Type II cases may also present with these conditions when communication with the pulp or periodontal tissues occurs [5, 9]. As a result, endodontic management is often required to preserve and treat the affected tooth [1].

The clinical presentation of DI varies significantly [2, 3, 10]. The crown of the affected tooth may exhibit normal morphology or display unaesthetic features and is often associated with other aesthetic issues [11]. A commonly recommended restorative protocol involves the fabrication of composite resin veneers, which do not require significant preparations such as vestibular, proximal, and incisal reduction [12]. This technique relies solely on proper acid conditioning of the enamel substrate for strength and retention [13].

This case report is aimed at describing the successful endoaesthetic management of a Type II DI in a maxillary lateral incisor with root perforation and an apical abscess.

## 2. Case Report

A 12-year-old female patient was referred to a specialized endodontic practice for the assessment and management of her maxillary right lateral incisor (Tooth 12). One week earlier, her general dentist diagnosed DI and, after an unsuccessful attempt to access the root canal, which caused a root perforation, referred her for further treatment. The patient, who was undergoing orthodontic treatment, reported pain and swelling in tooth 12 for the past 3 months. Additionally, she expressed concerns about the aesthetic appearance of her maxillary anterior teeth, specifically the presence of diastemas, which affected her smile.

The patient had no relevant medical history. Extraoral examination revealed no abnormalities. On clinical examination, Tooth 12 showed no abnormal anatomy on its clinical crown, and its color was normal. However, the palatal surface of Tooth 12 had been accessed and temporarily restored by the patient's general dentist. The tooth exhibited slight tenderness to direct vertical percussion but had normal mobility and responded normally to buccal and palatal palpation. Cold thermal tests (Roeko Endo Frost; Roeko, Hangenav, Germany) elicited negative responses for Teeth 12 and 13, while adjacent teeth responded normally. The patient reported no history of previous trauma.

**Figure 1:**
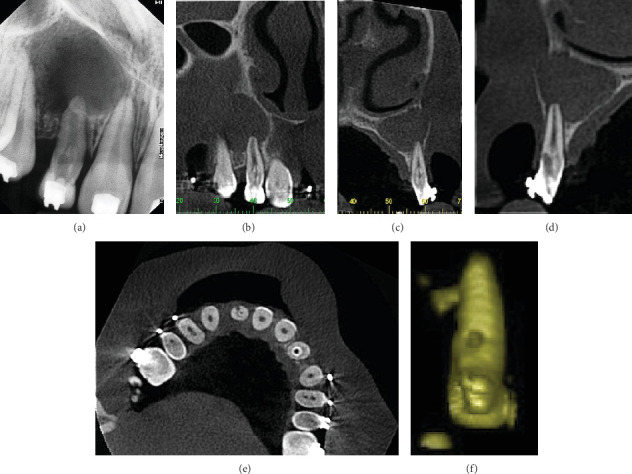
(a) Periapical radiographic examination of Tooth 12 confirming abnormal morphology and signs of prior endodontic access. (b, c) CBCT scans revealing a Type II invagination in the maxillary right lateral incisor and a well-defined periapical lesion around the apices of Teeth 11, 12, and 13. (d, e) CBCT scans of Tooth 12 showing excessive tooth wear and deviation of the root canal path toward the buccal surface. (f) 3D reconstruction confirming root perforation, illustrating the extent and location of the defect.

A periapical radiographic examination of Tooth 12 confirmed the presence of abnormal morphology and revealed signs of previous endodontic access. Additionally, an extended periradicular radiolucency was observed in the periapical area of Teeth 12 and 13 (Figure 1). To obtain more detailed information about the internal anatomy of the invaginated tooth, a CBCT scan was indicated. The CBCT images revealed a Type II invagination in both maxillary lateral incisors (Teeth 12 and 22), which penetrated the middle third of the root but remained confined as a blind sac. A well-defined periapical lesion, larger than that observed in conventional radiography, was evident around the apexes of Teeth 11, 12, and 13 (Figure 1). Furthermore, excessive tooth wear with a deviation of the root canal path toward the buccal surface, indicative of root perforation, was observed on Tooth 12 (Figures 1, 1, and 1).

The diagnosis of Oehler's Type II DI with root perforation and apical abscess was established for Tooth 12, and pulp necrosis was diagnosed for Tooth 13. The recommended treatment for Tooth 12 involved nonsurgical root canal treatment and repair of the root perforation. Similarly, nonsurgical root canal treatment was also advised for Tooth 13.

**Figure 2:**
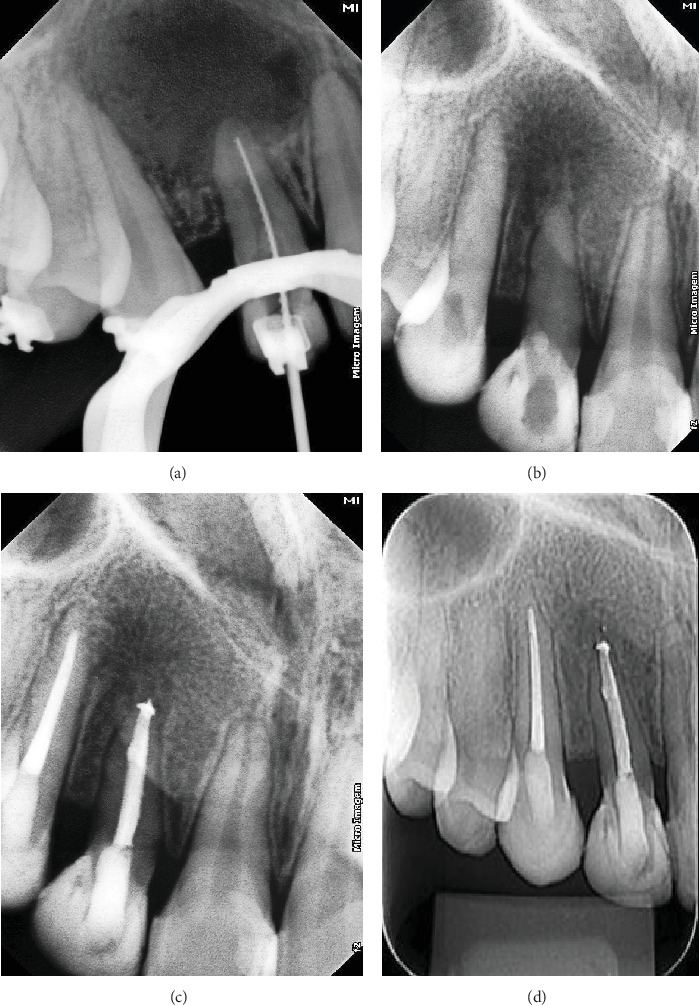
(a) The working length determination radiograph. (b) Application of calcium hydroxide as an intracanal dressing. (c) Final obturation of the root canal. (d) Follow-up visit 7 months later, demonstrating significance in the periapical radiolucency.

**Figure 3:**
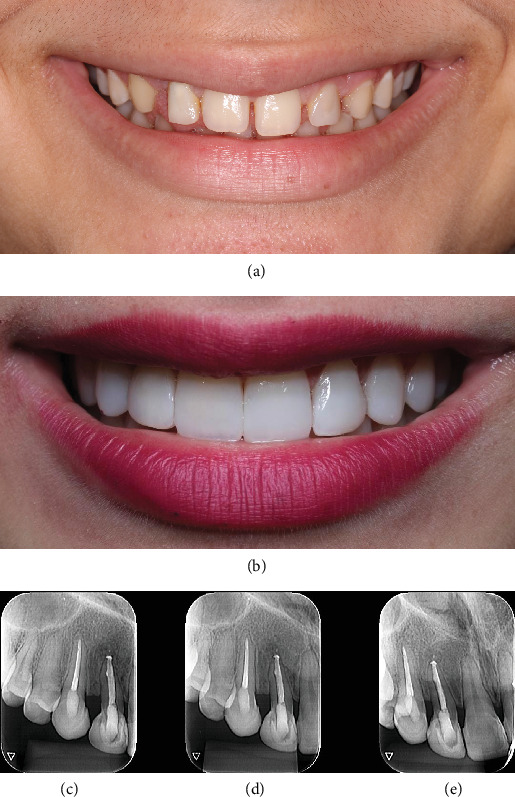
(a, b) Application of composite resin veneers on the upper anterior teeth to address the patient's aesthetic concerns. (c–e) Follow-up visit 2 years posttreatment showing complete resolution of the periapical lesion.

During the initial visit, the region around Tooth 12 was anesthetized using 2% lidocaine with 1:100,000 epinephrine (DFL Indústria e Comércio, Rio de Janeiro, Rio de Janeiro, Brazil). The temporary restorative material was removed using a round diamond bur (#1013; KG Sorensen, Barueri, São Paulo, Brazil). A persistent exudate was noted from the root canal, and the root perforation was promptly identified just above the gingival margin. To seal the perforation provisionally, glass ionomer cement (Maxxion R; FGM, Joinville, Santa Catarina, Brazil) was applied. In the sequence, a size 15 K-file (Dentsply Maillefer, Ballaigues, Switzerland) was used to explore the root canal. The working length was established using an apex locator (Propex II; Dentsply Maillefer), confirmed subsequently with a periapical radiograph (Figure 2). The canal was gradually prepared with the WaveOne Gold system (Dentsply Maillefer) up to the Large file (#45/05). Irrigation of the canal was performed using 2.5% sodium hypochlorite (NaOCl) (Fitofarma, Goiânia, Goiás, Brazil), followed by a final flush with 17% EDTA (Biodinâmica, Itaporã, Paraná, Brazil) and neutralization with 2.5% NaOCl. Activation of all irrigant solutions was achieved using an ultrasonic tip (E-1 irrisonic; Helse Technology, Santa Rosa do Viterbo, São Paulo, Brazil) at a Power Setting 1 of an ultrasonic unit (EMS PM 200; EMS-Electro Medical Systems, Nylon, Switzerland) to enhance the contact between the solution and the surface of the root canal walls. Following the final irrigation protocol, the canal was dried with sterile absorbent paper points (Dentsply Maillefer) and dressed with calcium hydroxide paste (Biodinâmica Química e Farmacêutica Ltda., Ibiporã, Paraná, Brazil). Subsequently, the cavity was restored with a provisional material (Maxxion R; FGM, Joinville, Santa Catarina, Brazil), and the patient was scheduled for a follow-up appointment in 2 weeks (Figure 3).

The patient returned for a follow-up appointment 2 weeks later to continue treatment. A surgical procedure was proposed for the definitive sealing of the perforation. Initially, a surgical flap was created to provide direct access to the perforation site. Considering the supraosseous location of the root perforation, composite resin (3 M ESPE, Saint Paul, Minnesota, Michigan, United States) was selected as the sealing material. After sealing, the flap was repositioned and sutured using Ethicon Nylon 5.0 (Johnson & Johnson do Brasil Indústria e Comércio de Produtos para Saúde Ltda., São Paulo, São Paulo, Brazil), ensuring proper tissue coverage and facilitating healing.

After 21 days, the endodontic treatment was resumed. Calcium hydroxide paste (Biodinâmica Química e Farmacêutica Ltda., Ibiporã, Paraná, Brazil) was applied initially every 2 weeks, then extended to monthly intervals, and eventually to every 2 months. Ten months after initiating treatment, no persistent exudate or evidence of periapical inflammation was observed, indicating readiness for root canal filling (Figure 2b). Removal of temporary restorative material was followed by irrigation and repreparation of the root canal, following the same protocol as the initial appointment. A master gutta-percha cone radiograph was obtained. Subsequently, the root canal was dried using sterilized absorbent paper points (Dentsply Maillefer) and filled using the lateral condensation technique with gutta-percha (Tanari, Manaus, Amazonas, Brazil) and an epoxy resin–based sealer (AH Plus; Dentsply Maillefer). The access cavity was finally restored with light-cured composite resin (3M ESPE, Saint Paul, Minnesota, Michigan, United States). A postoperative periapical image confirmed adequate root canal obturation (Figure 2d). Similar treatment procedures were conducted for Tooth 13, following the protocol used for Tooth 12.

The patient was scheduled for follow-up visits and was seen again after 7 months. The tooth was asymptomatic, and radiography confirmed a significant reduction in the size of the periapical radiolucency associated with Tooth 12 (Figure 2e). Consequently, the patient was referred for aesthetic treatment.

After endodontic treatment, in-office bleaching was performed, followed by the fabrication of composite resin veneers on the upper anterior teeth to address the patient's aesthetic concerns (Figure 3a). Considering the patient's age, the veneers were fabricated using the incremental method without any tooth preparation.

For the fabrication of the composite resin veneers, the preformed metal matrix technique was employed. Initially, following waxing on a gypsum model, a guide was crafted using Zetalabor laboratory condensation silicone (Zhermach, Badia Polesine, Italy). The bis-acryl composite resin Systemp C&B II (Ivoclar Vivadent, Schaan, Liechtenstein) was inserted into the internal portion of the guide and placed in the patient's mouth, establishing the mock-up. The patient assessed the mock-up and demonstrated a high degree of satisfaction and approval of the immediate provisional result.

With the approval of the provisional result, a new impression of the wax model was made using laboratory condensation silicone Zetalabor (Zhermach, Badia Polesine, Italy) to fabricate a new guiding template. After the setting of the impression material, the mold was removed and cut mesiodistally with a #12 surgical blade, removing only the vestibular portion and preserving the incisal edge to obtain the restorative matrix.

Next, the matrix was placed intraorally to verify and test the correct fit. Careful smoothing of the subgingival proximal surfaces was performed using diamond-coated strips, avoiding gingival bleeding, followed by the isolation of the operative field with a rubber dam. After isolation, the teeth's surfaces were conditioned with 37% phosphoric acid (Power Etching, BM4, Palhoça, Santa Catarina, Brazil) for 30 s, followed by thorough rinsing and drying with an air jet. Subsequently, the adhesive system Adper Single Bond (3M ESPE, St. Paul, Minnesota, United States) was applied and light-cured for 20 s.

The restorative stage began by applying Z350 XT resin (3M ESPE, St. Paul, Minnesota, United States), Shade WE, onto the matrix to create the palatal shell that would encompass the entire palatal space to be restored, leaving the incisal portion intact. The silicone matrix was positioned, and in the preserved incisal area, an opaque halo was created using Z350 XT resin (3M ESPE, St. Paul, Minnesota, United States), Shade WB. After polymerization, the matrix was removed.

For the proximal surfaces, a preformed metal matrix Unimatrix (TDV, Pomerode, Santa Catarina, Brazil) was used along with Z350 XT resin (3M ESPE, St. Paul, Minnesota, United States) in Shades A1B and WB. After restoring the proximal surfaces, the rubber dam was removed. Then, a layer of dentin and mamelon resin was applied to the vestibular surfaces, using Z350 XT resin (3M ESPE, St. Paul, Minnesota, United States) in Shades A1B and WB. A flow resin and Z350 XT resin (3 M ESPE, St. Paul, Minnesota, United States) in Shade GT were employed for the translucent halo, and BL-L Empress Direct enamel resin was used for the vestibular enamel. In the next session, the complete finishing and polishing process was carried out. An excellent aesthetic result was achieved (Figure 3b).

Follow-up examinations were scheduled to evaluate the case evolution. At a 2-year recall appointment, the patient was asymptomatic, and radiographic assessment demonstrated complete resolution of the periapical lesion (Figures 3c, 3d, and 3e).

## 3. Discussion

The successful management of Type II DI with associated root perforation and apical abscess, as described in this case, highlights the importance of accurate diagnostics, multidisciplinary collaboration, and advanced endodontic and restorative techniques.

Although conventional radiographic techniques are commonly used [1], they often fail to reveal the detailed anatomy of DI [2, 6, 7], which can result in misdiagnosis or incomplete treatment plans [7, 10, 11]. In this case, CBCT provided sagittal, coronal, and axial views, allowing a comprehensive evaluation of the invagination's depth, root canal anatomy, and the extent of the periapical lesion. This advanced imaging facilitated tailored treatment strategies, such as the precise localization of the root perforation and assessment of the lesion's progression, thereby reducing the risk of treatment failures [1, 5].

Type II DI, characterized by an enamel-lined invagination extending into the root and forming a blind sac [8], often complicates instrumentation, disinfection, and obturation [3, 4]. In the present case, the presence of a root perforation further increased the complexity. Root perforations, whether congenital or iatrogenic, compromise the treatment prognosis and require careful management to prevent microbial infiltration and subsequent failure [11]. The chosen approach involved a combination of nonsurgical root canal treatment and root perforation sealing using composite resin. Calcium hydroxide was used as an intracanal dressing for an extended period, addressing persistent exudation, and promoting healing [10, 14]. This material's well-documented antimicrobial properties and ability to neutralize endotoxins are instrumental in resolving the periapical lesion [14]. The use of ultrasonic activation of irrigants, including 2.5% NaOCl and 17% EDTA, enhanced the cleaning of the root canal system, particularly in inaccessible areas, as evidenced by the literature [2, 5]. These advanced disinfection protocols significantly improved the likelihood of successful treatment in such a complex anatomical scenario [4, 5].

The multidisciplinary nature of the treatment ensured not only the resolution of the periapical pathology but also the restoration of aesthetics, which was a primary concern for the patient. After the successful completion of endodontic therapy, a minimally invasive restorative approach was adopted using direct composite resin veneers. Preformed metal matrices were employed to enhance the precision and longevity of the restorations while minimizing tooth structure loss [12]. Composite resin veneers were selected due to their conservative preparation requirements, which preserve the enamel substrate, an essential factor for achieving strong adhesion and durable results [13]. The use of a preformed matrix simplified the fabrication of proximal surfaces, reducing operator variability and optimizing the aesthetic outcome [12].

This case emphasizes the critical importance of accurate diagnosis and timely referral in managing DI. The lack of initial recognition and suboptimal treatment by the general practitioner led to complications such as root perforation. A previous study has shown that only a minority of general dentists feel confident managing such complex cases and often fail to refer them to specialists [15]. This highlights the need for increased awareness and education about developmental dental anomalies among practitioners [4].

## 4. Conclusion

This case underscores the importance of multidisciplinary collaboration, precise treatment planning, and patient-centered care in achieving optimal endoaesthetic results in complex cases.

## Data Availability

The data that support the findings of this study are available from the corresponding author upon reasonable request.
